# Demonstration of High-Power and Stable Single-Mode in a Quantum Cascade Laser Using Buried Sampled Grating

**DOI:** 10.1186/s11671-019-2954-6

**Published:** 2019-04-03

**Authors:** Feng-Min Cheng, Jin-Chuan Zhang, Dong-Bo Wang, Zeng-hui Gu, Ning Zhuo, Shen-Qiang Zhai, Li-Jun Wang, Jun-Qi Liu, Shu-Man Liu, Feng-Qi Liu, Zhan-Guo Wang

**Affiliations:** 1 Key Laboratory of Semiconductor Materials Science, Beijing Key Laboratory of Low Dimensional Semiconductor Materials and Devices, Institute of Semiconductors, Chinese Academy of Sciences, P.O. Box 912, Beijing, 100083 China; 2 Center of Materials Science and Optoelectronics Engineering, University of Chinese Academy of Sciences, Beijing, 100049 China

**Keywords:** Quantum cascade laser, Distributed feedback, Sampled grating

## Abstract

High-power, low-threshold stable single-mode operation buried distributed feedback quantum cascade laser by incorporating sampled grating emitting at *λ* ~ 4.87 μm is demonstrated. The high continuous wave (CW) output power of 948 mW and 649 mW for a 6-mm and 4-mm cavity length is obtained at 20 °C, respectively, which benefits from the optimized optical field distribution of sampled grating. The single-mode yields of the devices are obviously enhanced by controlling cleaved positions of the two end facets precisely. As a result, stable single-mode emission and mode tuning linearly without any mode hopping of devices are obtained under the different heat sink temperatures or high injection currents.

## Introduction

Quantum cascade lasers (QCLs) have turned out to be one of the most promising mid-infrared light sources and attracted much attention for the applications of remote sensing, high-resolution spectroscopy, and industrial process monitoring after its first demonstration due to its highlight features such as large wavelength covering range, compact size, and high output power [1–4]. As for those applications, single-mode emission and high output power are usually desired, which can be achieved by a distributed feedback (DFB) QCL. The buried grating approach has been adopted widely for a smaller waveguide loss, lower threshold current density, and higher single-mode yields compared with the surface grating [5, 6]. Up to now, a series of significant breakthroughs based on a buried grating approach have been made in improving the performance of DFB QCLs of single-mode stability and output power [7, 8], but an over-coupled feedback mechanism of buried grating hinders the output power from enhancing further. The typical value of continuous wave (CW) output power of buried uniform grating DFB QCLs emitting around 4.6–5 μm is less than 300 mW at room temperature [5, 9]. Theoretically, the coupling coefficient of buried grating can be improved by optimizing the grating depth and the duty cycle. However, the distributed feedback performance levels are very sensitive to the profile of etching of grating in the semiconductor layer close to the active area. Any tiny variation of the etching depth and the duty cycle would strongly influence the grating coupling coefficient [10, 11]. Moreover, it is also difficult to improve the grating coupling by controlling the grating depth and the duty cycle precisely based on a low-cost holographic lithography technique and wet chemical etching. Generally, the conventional DFB QCLs oscillate at two frequencies slightly shifted from the Bragg frequency, which can lase depending on the optical loss influenced by the facet random phase [12–14].

In this work, we propose the use of buried sampled grating with a small sampling duty cycle for optimizing the coupling coefficient and improving the optical field distribution. The prominent advantage of this method is it is able to increase the cavity length of device for enough optical gain while maintaining a desirable grating coupling strength. To improve the single-mode yields and ultimate performance, cleaved position of the two end facets is precisely controlled to avoid the effect of the end facet random phase. On the one hand, this approach retains the advantages of small waveguide loss for a low threshold current density and is compatible with buried heterostructure processing. Furthermore, the sampled grating is fabricated only through conventional holographic exposure combined with the optical photolithography, which leads to improved flexibility, repeatability, and cost-effectiveness. As a result, low threshold and high-output power single-mode DFB QCLs emitting at *λ* ∼ 4.87 μm are achieved simultaneously in the buried sampled grating structure. The threshold current density of these DFB-QCLs is as low as 1.05 kA/cm^2^ and the single facet produced 948 mW of CW output power for the device with a 6-mm cavity length at 20 °C.

## Methods

**Fig. 1:**
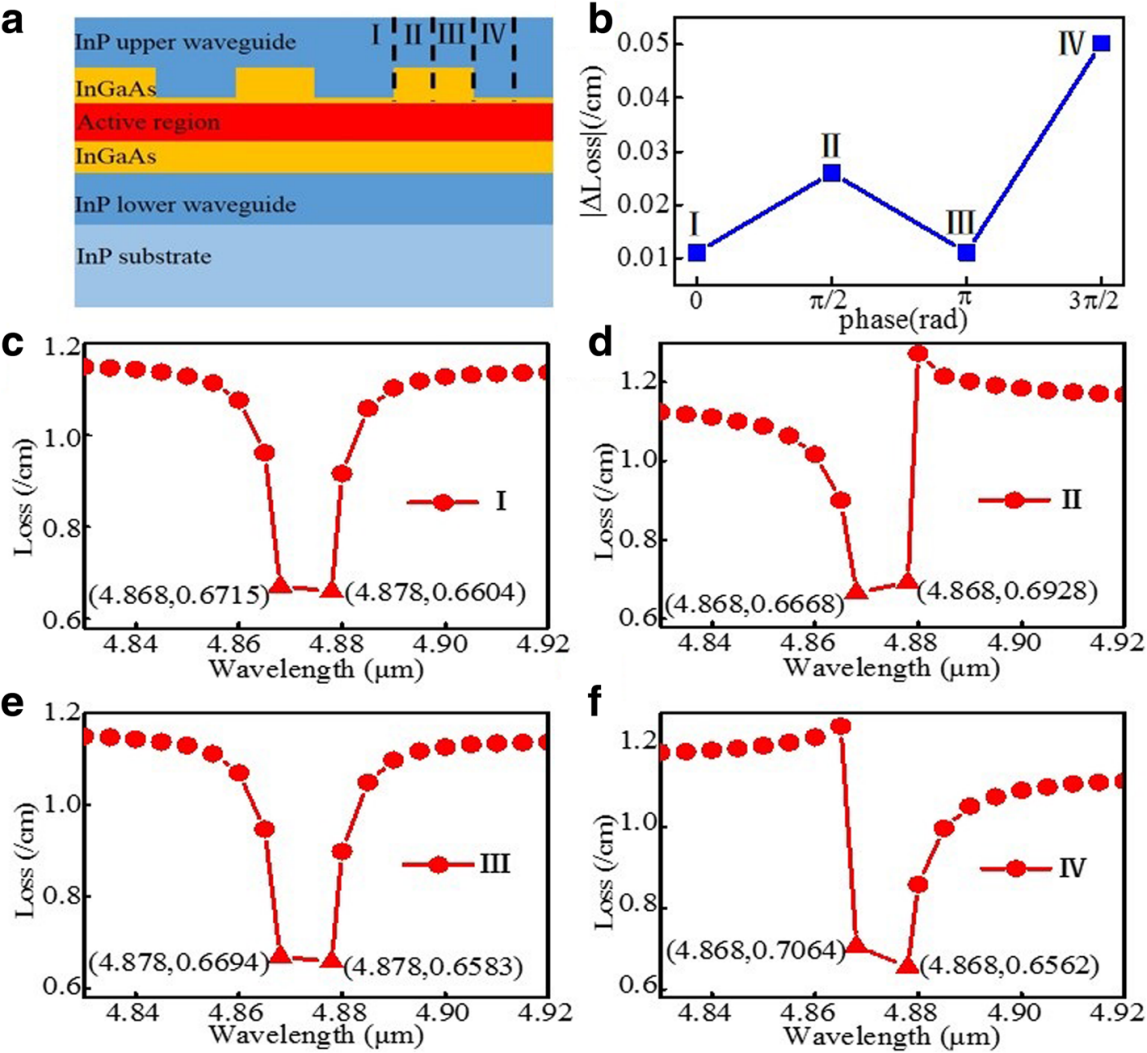
**a** The diagram of uniform grating structure; the denotations of I, II, III, and IV represent the possible four kinds of cleaved end facet positions. **b** The absolute values of mode loss difference for different cleaved end facet positions of I, II, III, and IV, and the abscissa is represented as a corresponding phase of cleaved facet positions of I, II, III, and IV. **c**–**f** The spectra of mode losses of the possible four kinds of cleaved end facet positions of I, II, III, and IV, respectively

**Fig. 2:**
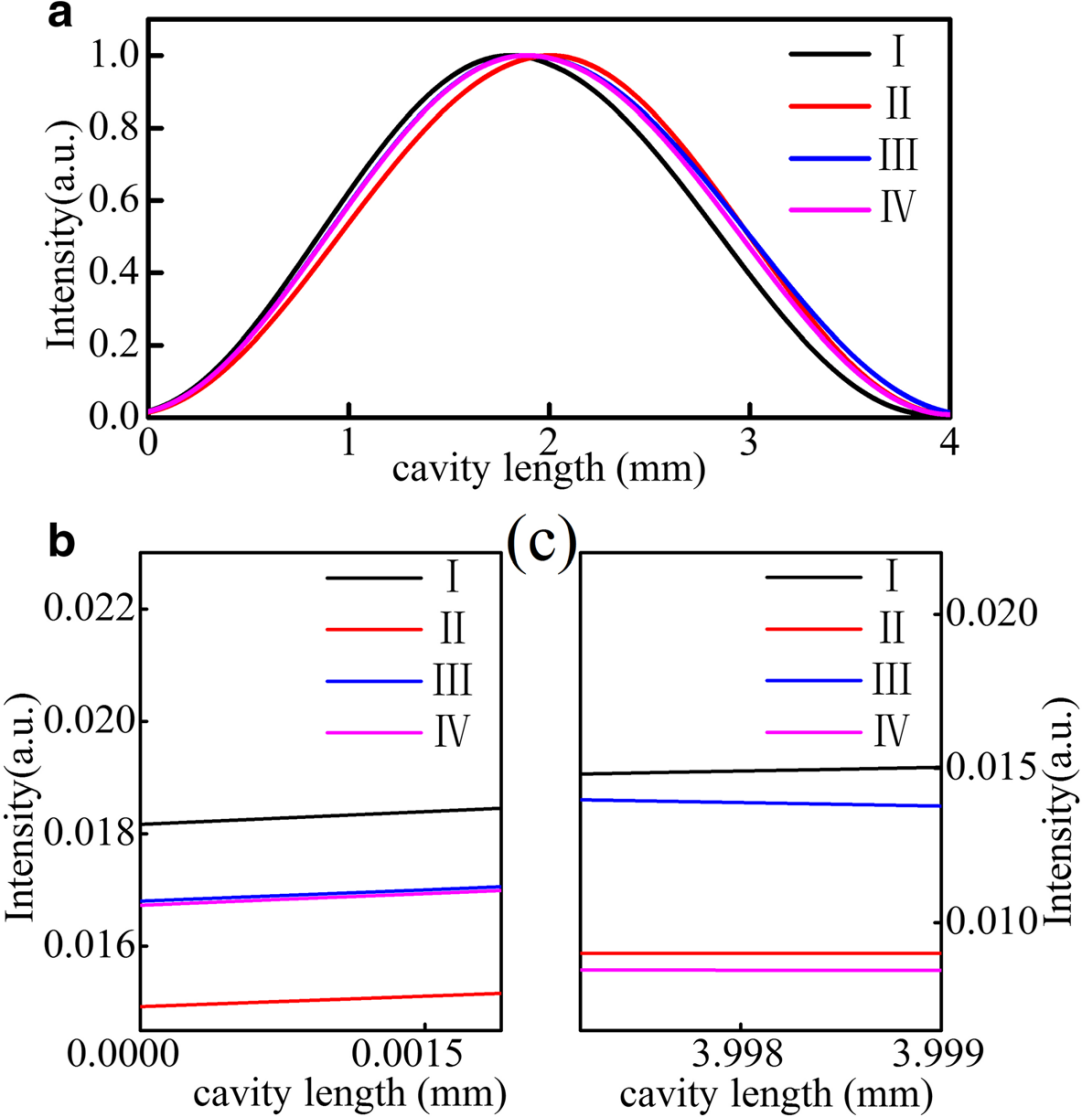
**a** The corresponding optical field distribution of the uniform grating structure for the four kinds of cleaved end facet positions of I, II, III, and IV. **b**, **c** The amplification of the optical field distribution near the two end facets

A diagram of the uniform grating DFB QCL is shown in Fig. 1a; the marks of I, II, III, and IV represent the possible four kinds of cleaved end facet positions. As we all know, it is difficult to control precisely the cleaved facet position for nanoscale uniform grating. As a result, the emission mode is different from device to device for the cleaved facet position is random. Here we simulate and calculate the loss difference of the two side modes and spectra of mode losses of possible four kinds of cleaved end facet positions of I, II, III, and IV based on the transfer matrix method by MATLAB. The absolute value of loss difference of the two side modes of the four kinds of cleaved end facet positions of I, II, III, and IV is shown in Fig. 1b; the abscissa is represented as the relative position of I, II, III, and IV (assuming that another facet begins with just the grating peak and it corresponds to the phase of zero, then the corresponding phases of I, II, III, and IV are 0, π/2, π, and 3π/2). Figure 1c, d, e, and f show the spectra of mode losses of the four kinds of cleaved end facet positions in detail. As ones have seen, the lasing mode and loss difference are different from devices to devices influenced by the facet random phase. Figure 2a shows the corresponding normalized optical field distribution of possible four kinds of cleaved end facet positions of I, II, III, and IV simulated by the same transfer matrix method. Figure 2b and c are the amplification of the optical field distribution near the two end facets. As we have seen, the intensity of both end facets is not completely symmetric, which is caused by an asymmetric position of both end facets. Here we show the situation with coupling strength *κ* × *L* = 17, which is over-coupled. The light intensity peaks in the center of the device decays rapidly towards the two ends, which could lead to the severe spatial hole burning, and in turn, maintaining stable single-mode operation may become difficult [15].

**Fig. 3:**
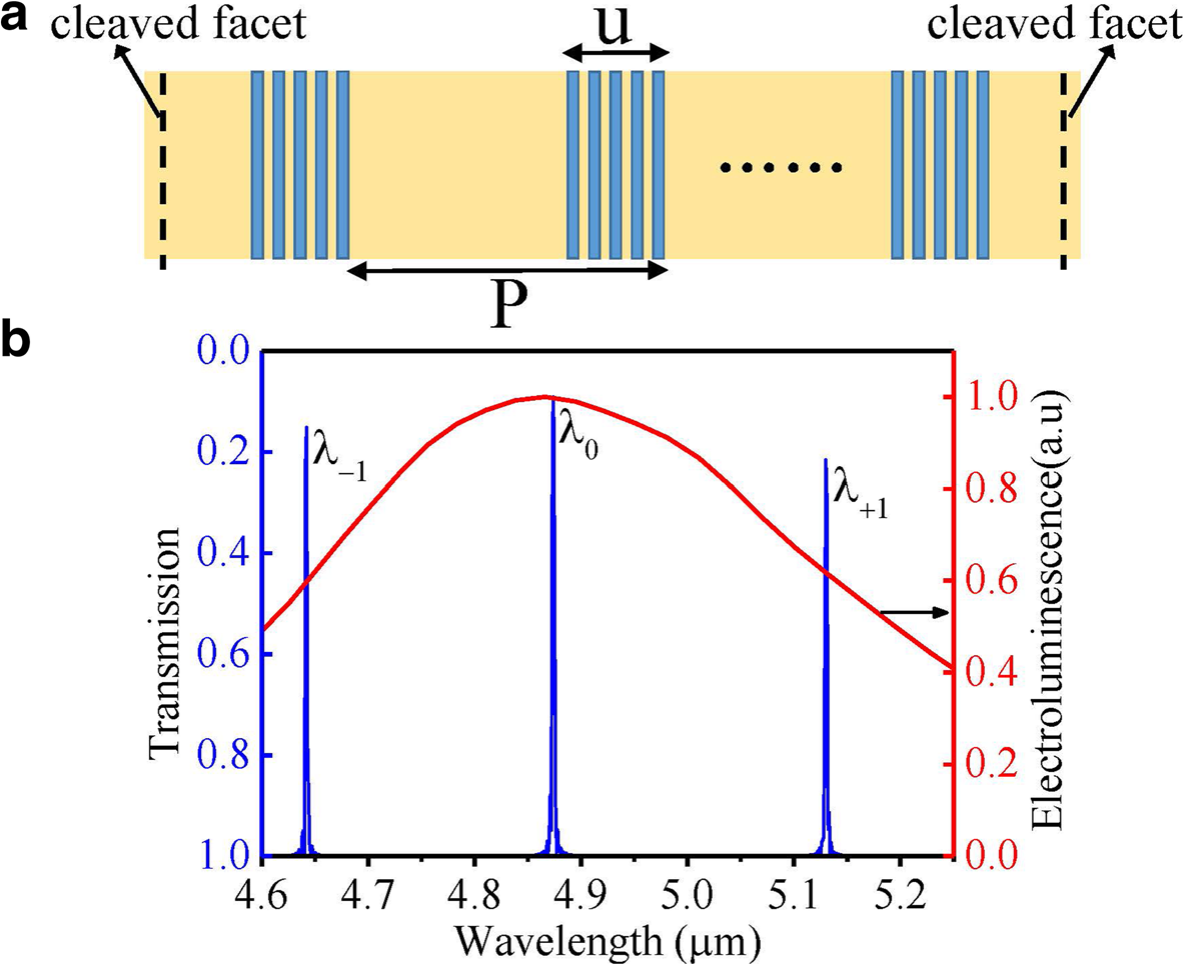
**a** The diagram of the sampling grating structure, the vertical dotted line represents the cleaved facet position, *P* is the sampling period, and *u* is the length of the grating region in one sampling period. **b** The blue line is the calculated transmission spectrum of the designed sampled grating, and the red line is the measured electroluminescence spectrum of the fabricated wafer

**Fig. 4.**
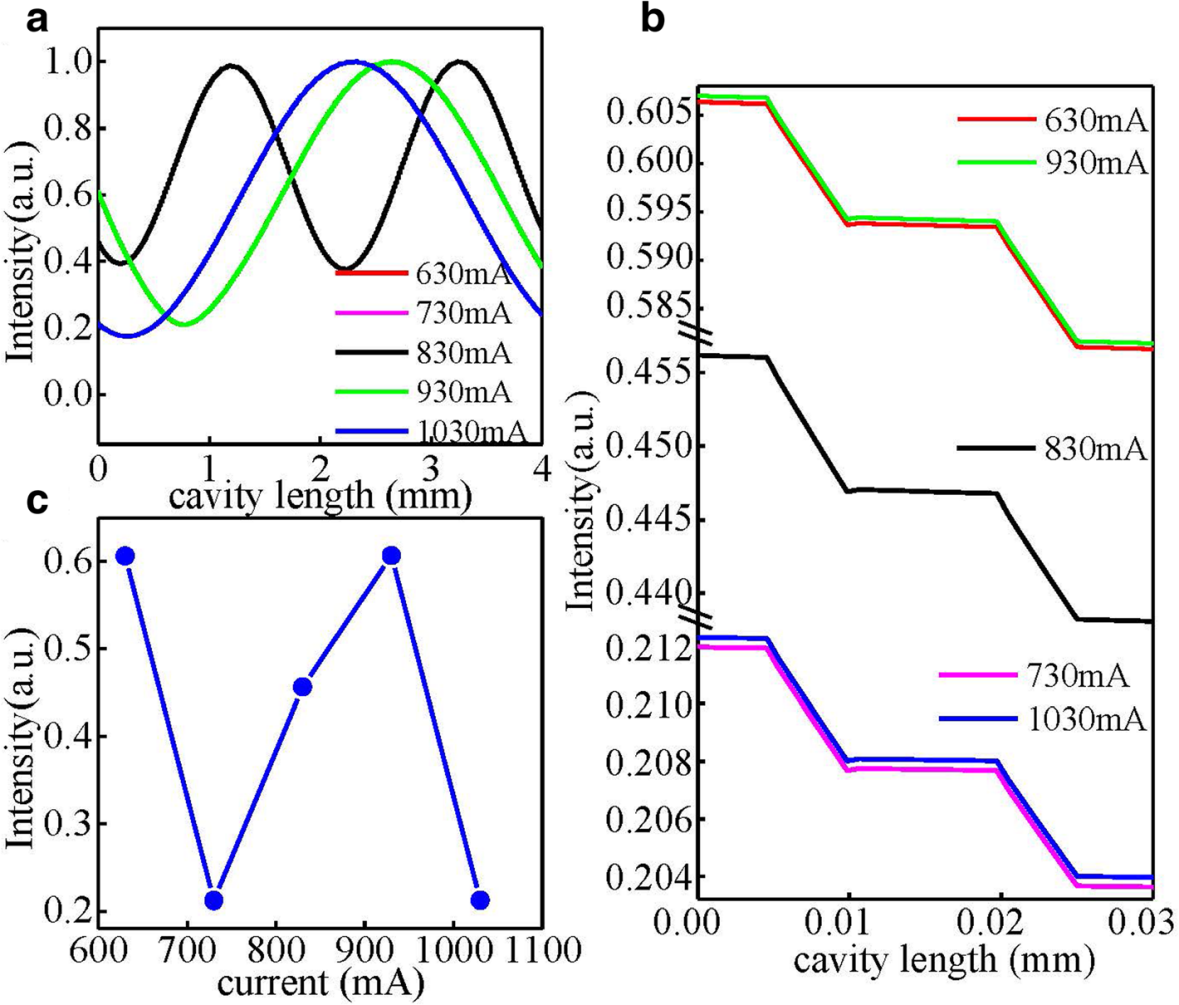
**a** The simulated optical field distribution of the sampling grating at different injection currents. **b** The amplification of the optical field distribution near one of the end facets. **c** The detailed variation of optical field relative intensity at the end facet with injection currents

Here we take a measure of the sampling grating structure to improve the distribution of optical intensity based on the sampling period of *P* = 15 μm and a block length of *u* = 5 μm corresponding to the sampling duty cycle of *σ* = *u*/*P* = 1/3, which is shown in Fig. 3a. The vertical dotted line of Fig. 3a represents the cleaved facet position, which deviates from the block region to avoid the introduction of the end facet random phase. The corresponding effective coupling coefficient *κ*_eff_ can be simply given by the product of the coupling coefficient *κ* of the uniform grating times the duty cycle *σ* of the sampling grating, that is, *κ*_eff_ = *κ* × *σ* [16]. So the coupling coefficient of the sampling grating could be adjusted arbitrarily by the duty cycle of the sampled grating, which is to the benefit of optimizing the coupling strength of the sampling grating. As a result, the output power could be improved. Figure 3b shows the calculated transmission spectrum of sampled grating based on the transfer matrix method and the measured electroluminescence (EL) spectrum under pulsed condition. The *λ*_−1_ and *λ*_+1_ are the additional super-modes introduced by the sampled grating. The adjacent spectral distance of super-modes can be calculated by Δ*λ* = *λ*_B_^2^/(2*n*_eff_*P*) where *n*_eff_ is the effective index of the waveguide and *λ*_B_ is the Bragg wavelength of the basic uniform grating [17]. Although the existence of super-modes may influence the single-mode stability, the super-modes can be designed far away from the gain curve center by choosing a small sampling period *P* according to the formula of spectral distance of super-modes. In our study, the basic Bragg grating period *Λ*, sampling period *P*, effective index of the waveguide *n*_eff_, and duty cycle *σ* are 0.758 μm, 15 μm, 3.21, and 1/3, respectively, so the adjacent spectral distance of super-modes is around 246 nm. As Fig. 3b shows, the Bragg wavelength (0th order) is designed around the peak of the gain curve, while the + 1st- and − 1st-order wavelength are 246 nm away from the gain curve center separately. Finally, stable single-mode lasing at the 0th order mode in our study can be achieved. Figure 4a shows the simulated optical field distribution of the sampling grating at different injection currents. As can be seen, there has been a dramatic improvement in the optical field intensity distribution for the sampling grating structure at the two end facets, which corresponds to a major improvement in output power. Figure 4b is an amplification of the optical field distribution near one of the end facets, and Fig. 4c displays the detailed variation of the optical field intensity at the end facet with injection currents. As shown in Fig. 4b, the profile of the optical field distribution is not smooth but fluctuant. The fluctuation is caused by the “interface reflection” between the block region and non-grating region in each sampling period inducing a “localized” energy density concentration along the cavity. Besides, as shown in Fig. 4c, the variation of relative intensity distribution of the end facet is nonlinear with the injection currents, which may cause nonlinearity in the power–current curve of devices.

The QCL structure was grown on an n-InP (Si, 2 × 10^17^ cm^−3^) substrate by solid-source molecular beam epitaxy (MBE). The active core consisted of 40 stages of strain-compensated In_0.67_Ga_0.33_As/In_0.37_Al_0.63_As quantum wells and barriers providing the electron transition channel to produce photon, which was surrounded by the upper and lower InGaAs confinement layers. The grating was defined on the upper InGaAs confinement layer using a double-beam holographic lithography technique combined with conventional optical lithography. Then the upper waveguide layer was grown by metal organic vapor phase epitaxy (MOVPE). After that, the wafer was processed into a double-channel ridge waveguide laser with an average core width of about 10 μm filling with semi-insulating InP:Fe for efficient heat removal. A 450-nm-thick SiO_2_ layer was then deposited by plasma-enhanced chemical vapor deposition (PECVD) for insulation, and electrical contact was provided by a Ti/Au layer deposited by electron beam evaporation. An additional 5-μm-thick gold layer was electroplated for improving heat dissipation. After being thinned down to about 140 μm, a Ge/Au/Ni/Au metal contact layer was deposited on the substrate side. Then the waveguides were cleaved into 4-mm- and 6-mm-long bars, and the high reflectivity (HR) coating consisting of Al_2_O_3_/Ti/Au/Al_2_O_3_ (200/10/100/120 nm) was deposited on one of the facets by electron beam evaporation, leaving an uncoated facet for the measurement of edging emitting power. Lastly, the lasers were mounted with the epilayer side-down on a diamond heat sink with an indium solder, which were subsequently soldered on copper heat sinks for effective heat dissipation.

## Results and Discussion

The spectra of devices were tested by a Fourier transform infrared spectrometer with a resolution of 0.25 cm^−1^. The lasers were then mounted on a holder containing a thermistor combined with a thermoelectric cooler to monitor and adjust the sub-mount temperature. The emitted optical power was measured with a calibrated thermopile detector placed in front of the laser facet without any correction.

**Fig. 5:**
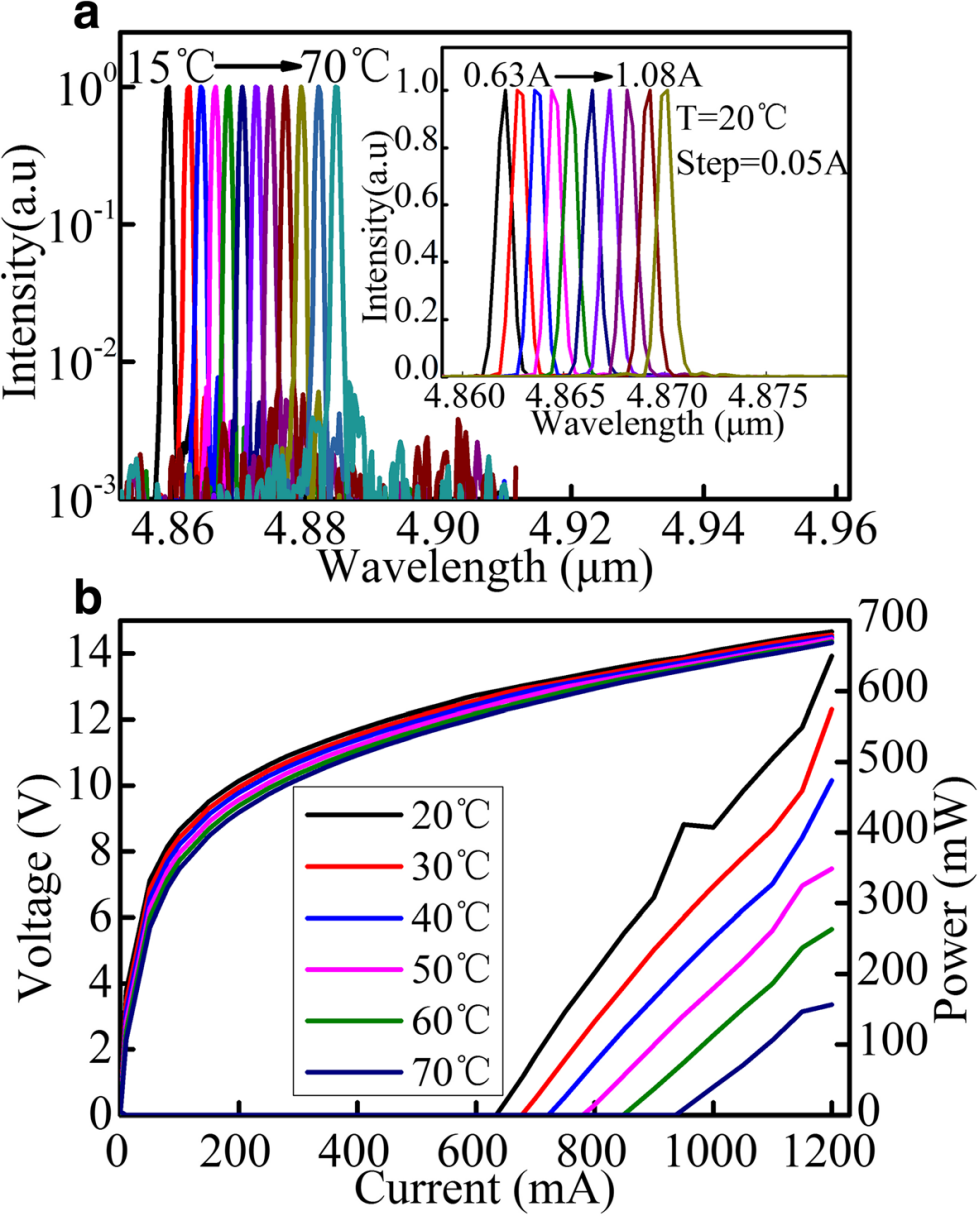
**a** Single-mode CW emission spectra of a sampled grating DFB QCL with the cavity length of 4 mm at currents of about 1.1 × *I*_th_ for different heat sink temperatures of 15–70 °C. The inset shows CW emission spectra at different injection currents from 0.63 to 1.08 A with a step of 0.05 A at 20 °C. **b** CW light–current–voltage (L–I–V) characteristics of sampled grating DFB QCL with the cavity length of 4 mm at different temperatures

**Fig. 6:**
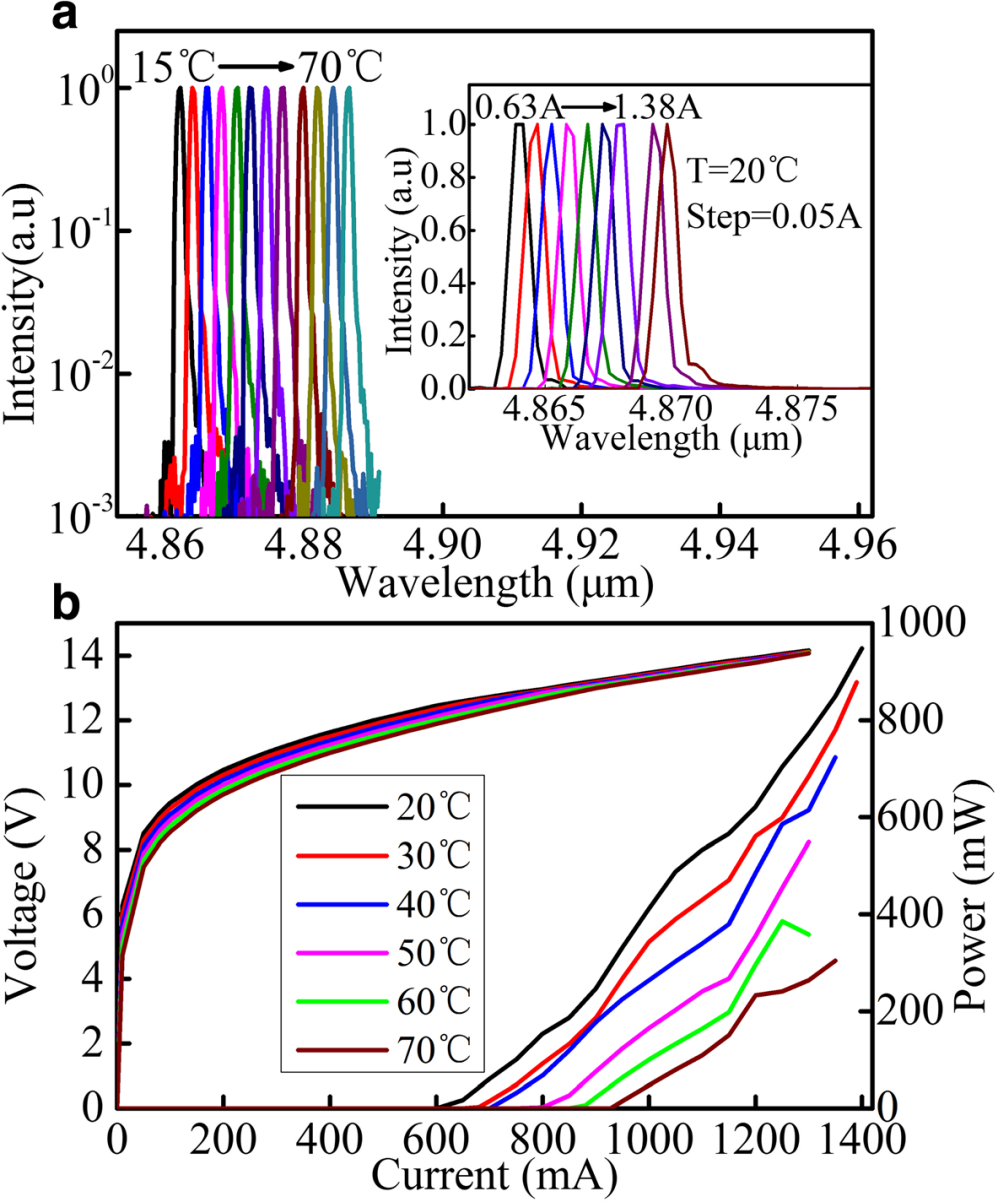
**a** Single-mode CW emission spectra of a sampled grating DFB QCL with the cavity length of 6 mm at currents around 1.1 × *I*_th_ for different heat sink temperatures of 15–70 °C. The inset shows CW emission spectra at different injection currents from 0.63 to 1.38 A with a step of 0.05 A at 20 °C. **b** CW light–current–voltage (L–I–V) characteristics of sampled grating DFB QCL with the cavity length of 6 mm at different temperatures.

Figures 5 and 6 show the emission spectra and light–current–voltage (L–I–V) characteristics of the devices with a 4-mm and 6-mm cavity length sampled grating DFB QCLs, respectively. As ones have seen, the spectra vary linearly with the injection current or temperature during all test processes. In CW mode, the maximum optical power of devices is 649 mW and 948 mW at 20 °C for a 4-mm and 6-mm cavity length at 1.2 A and 1.4 A, respectively. In addition, the low CW threshold current density of devices of 1.59 kA/cm^2^ and 1.05 kA/cm^2^ at 20 °C for a 4-mm and 6-mm cavity length is achieved, which fully reflects the advantage of small waveguide loss and low threshold current density of buried grating. As we have observed from the lasing spectra, the lasing mode is linear with the changes of temperature or injection current, which indicates that mode hopping does not happen in the course of the change of injection current or temperature. However, the power–current curves are not linear, which is caused by the fluctuations of the optical field distribution of the sampling grating structure and the nonuniform change optical field intensity of the end facets with the injection currents analyzed before.

**Fig. 7:**
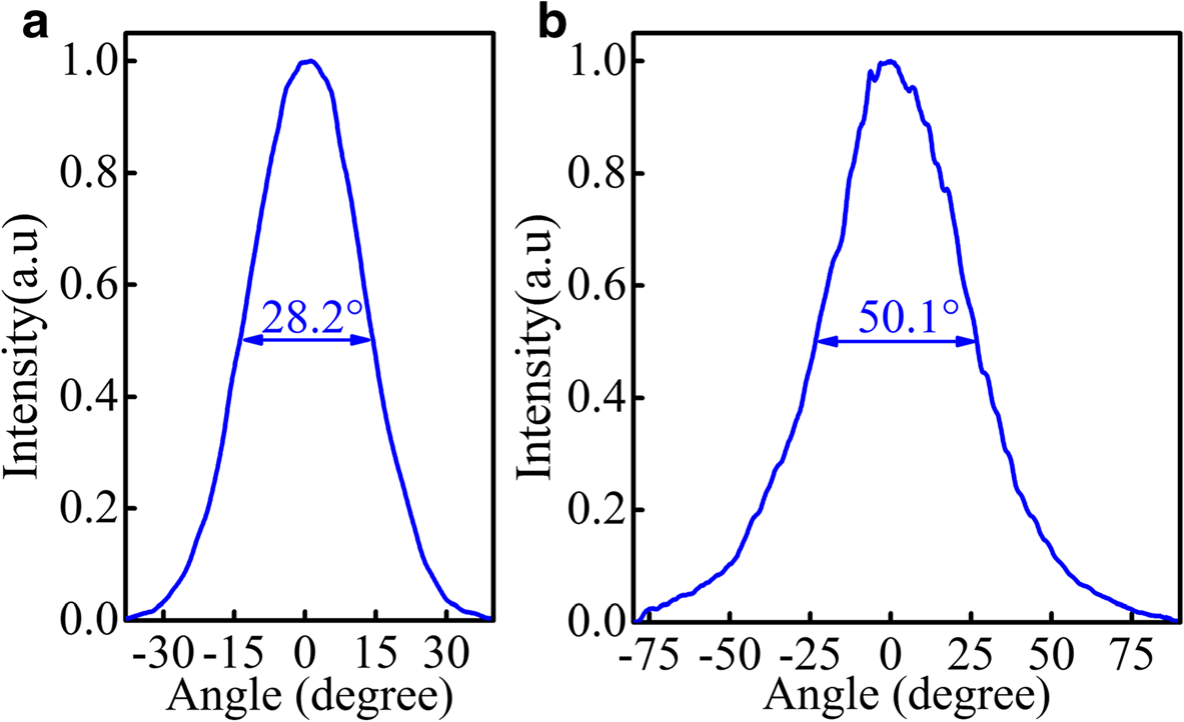
**a** The far-field profile along the ridge-width direction. **b** The far-field profile along the epitaxial growth direction

Figure 7 shows the far-field profiles of the device at pulsed operation about 1.25 × *I*_th_ at room temperature. Figure 7a shows the far-field profile along the ridge-width direction, and Fig. 7b displays the far-field profile along the epitaxial growth direction. Experimental studies demonstrated a fundamental transverse mode could more easily become the lasing mode in a buried grating structure than in a surface metal grating structure because the loss of fundamental transverse mode increases due to the coupling between fundamental transverse mode and the top metal contact in a surface metal grating structure [6]. According to that, the far-field profile of the fundamental transverse mode with the full width at half maximum (FWHM) of 28.2° along the direction of ridge-width has been obtained in our experiment. So another obvious advantage of buried grating conformation is displayed that lasing mode is generally a fundamental transverse mode with a single-lobe far-field profile, which is in favor of collimation. Additionally, a large FWHM of 50.1° along the epitaxial growth direction is obtained due to small emission aperture that is of the same order as the wavelength.

## Conclusions

In conclusion, low-threshold, high-output power stable single-mode emission sampling grating DFB QCLs have been achieved. The maximum CW output power and threshold current density are 0.948 W (0.649 W) and 1.05 kA/cm^2^ (1.59 kA/cm^2^) for a 6-mm (4 mm) cavity. A major improvement in distribution of the optical field is realized by introducing the small sampled duty cycle to reduce the coupling strength. A single lobe far-field profile is also observed. So for buried distributed feedback quantum cascade lasers, incorporating sampled grating is a simple and effective method to achieve the devices with high-output power, low-threshold, stable single-mode emission and high single-mode yields.

## Abbreviations

CW: Continuous wave
DFB: Distributed feedback
EL: Electroluminescence
FWHM: Full width at half maximum
HR: High reflectivity
L–I–V: Light–current–voltage
MBE: Molecular beam epitaxy
MOVPE: Metal organic vapor phase epitaxy
PECVD: Plasma-enhanced chemical vapor deposition
QCL: Quantum cascade laser
